# MultiTex RCT – a multifaceted intervention package for protection against cotton dust exposure among textile workers – a cluster randomized controlled trial in Pakistan: study protocol

**DOI:** 10.1186/s13063-019-3743-3

**Published:** 2019-12-16

**Authors:** Asaad Ahmed Nafees, Sara De Matteis, Muhammad Masood Kadir, Peter Burney, David Coggon, Sean Semple, Paul Cullinan

**Affiliations:** 1 Department of Community Health Sciences, Aga Khan University, Stadium Road, PO Box 3500, Karachi, 74800 Pakistan; 2 Population Health and Occupational Disease, National Heart and Lung Institute (NHLI), Imperial College London, London, UK; 3 MRC Lifecourse Epidemiology Unit, University of Southampton, Southampton, UK; 4 Institute for Social Marketing and Health Research, University of Stirling, Stirling, Scotland, UK

**Keywords:** Randomized controlled trial, Textile industry, Cotton fiber, Byssinosis

## Abstract

**Background:**

In the Pakistani textile industry the prevalence of workplace respiratory illnesses, including byssinosis, is high. The MultiTex RCT study aims to determine the effectiveness of a multifaceted intervention package in reducing dust levels in cotton mills, decreasing the frequency of respiratory symptoms among cotton textile workers, and improving their lung function.

**Methods/design:**

We will conduct a cluster-randomized controlled trial at 28 textile mills in Karachi. The intervention will comprise: training in occupational health for all workers and managers reinforced by regular refresher sessions; the formation of workplace committees to draw up, agree and promote a health and safety plan that includes wet mopping, safe disposal of cotton dust, and the use of simple face-masks, as well as further publicity about the risks from cotton dust; and provision of adequate supplies of face-masks to support the health and safety plan. Participating mills will be randomized to intervention and control arms following a baseline survey. The impact of the intervention will be determined through follow-up surveys conducted at 3, 12 and 18 months. Data collection in the surveys will include spirometry, questionnaire-based interviews and cotton-dust measurements.

**Discussion:**

If successful, the study may pave the way for simple, low-cost interventions that can help reduce cotton-dust levels in textile mills, and improve the respiratory health of textile workers in developing countries such as Pakistan.

**Trial registration:**

ClinicalTrials.gov, ID: NCT03738202. Registered on 12 November 2018.

## Background

Cotton is an important industrial crop, its production covering approximately 2.5% of the world’s arable land, and its processing providing employment to millions of people. These include those who work in plantations or in allied industries including textile manufacturing [1]. Pakistan is the fourth largest producer of cotton and the eighth largest exporter of textile products in Asia. Its textile industry accounts for 8.5% of national Gross Domestic Product (GDP) and employs around 40% of the industrial workforce [2–4]. In cotton mills the processes of ginning, spinning and weaving generate large quantities of “cotton dust” – a complex, organic mixture of ground-up plant matter, cotton fibers, bacteria, fungi, soil, pesticides, non-cotton matter and other contaminants. Gram-negative bacterial endotoxins in cotton dust are hypothesized to be an etiological agent for byssinosis, the disease classically associated with cotton-dust exposure among textile workers [5, 6], which is characterized by respiratory symptoms and lung function impairment [6]. Estimates of the prevalence of byssinosis among textile workers in Pakistan have ranged from 11 to 15% [7–9].

Several studies have demonstrated a significant dose-response relationship between cotton-dust exposure and byssinosis [6, 10]. Consequently, it seems likely that reductions in dust levels could improve the lung function of cotton mill workers [11, 12], with benefits apparent after less than a year [12]. Various measures have been tried to reduce the airborne levels of endotoxin in textile mills [13–16], but those which compromise the quality of yarn are generally not acceptable [14, 17]. Efforts have, therefore, focused mainly on attempts to reduce overall levels of dust exposure through strict enforcement of workplace exposure limits (WEL).

Engineering controls are the most effective way of reducing dust levels, but are relatively costly. Where they cannot be afforded, standard respirators (N95 and others) are recommended for cotton mill workers [18], but access to them has been limited in developing countries such as Pakistan. While there is little direct evidence regarding the effectiveness of such respirators and face-masks for protection against specific hazards [19], it is likely that they will provide some protection. In addition, training in occupational health and safety (OHS) may be beneficial, although the effectiveness of different learning approaches in such training is unknown [18, 20]. Thus, where engineering controls are not practicable, the best opportunity for the prevention of byssinosis may lie in a combination of various training methods with appropriate administrative controls and provision of face-masks. However, there is first a need for robust epidemiological assessment of such interventions, including through trials [20]. Few trials have been undertaken to test interventions to reduce occupational exposures; recent work by Basinas et al., for example, has shown that education and exposure feedback can reduce dust exposures of farmers by about 25% [21].

Building on an earlier pilot investigation [22], the study described in this paper is a randomized trial to assess the effectiveness of a multifaceted intervention package in reducing dust levels in the spinning and weaving sections of textile mills, decreasing the prevalence of respiratory symptoms associated with cotton-dust exposure among textile workers, and improving their lung function. As a secondary objective, it also aims to determine the effectiveness of the intervention package in reducing sickness absence attributed to respiratory disease.

## Methods/design

### Study design

A parallel, cluster-randomized controlled trial will be conducted with mills as the unit of randomization. After a baseline survey, the intervention package will be rolled out in mills allocated to the intervention arm, and its impact will be assessed through three follow-up surveys carried out across all mills. Analyses of effectiveness will be supplemented by a limited economic evaluation of the intervention, as described later.

### Recruitment and randomization

In Pakistan, no official sampling frame of textile mills is available but several business and textile associations represent the country’s textile mills and from them we will acquire a list of industrial plants in Karachi. To be eligible, textile mills will need to: (1) be from the formal sector and registered with business associations and the Government Labor Department; (2) have either spinning or weaving sections, or both; (3) employ at least 50 eligible workers in their spinning and weaving sections; (4) have a workforce in which at least 75% of staff have been employed for 12 months or longer; (5) have a management willing to participate in the planning and implementation of the study and (6) have no plans to introduce major improvements over the next 2 years.

Eligible workers at each mill will be identified by the Human Resources Department. We will include male textile workers aged ≥ 18 years who are employed in the bale-opening, blowing, carding, spinning, twisting, winding, warping, weaving or waste recycling sections. In Pakistan, women very rarely work in these sections and we will not include them. Those who do not give consent will be excluded. We will also exclude workers from the wet-processing areas (dyeing or bleaching), stitching and packaging sections, as well as support or administrative staff. At each mill, personal air sampling will be done on five purposively selected workers (100–150 in all) representing the range of sections and jobs. For this purpose, the latter will be categorized into one of four groups: (1) helpers, cleaners and doffers; (2) machine operators; (3) jobbers and fitters and (4) masters, in-charge or supervisors.

We will purposively select 28 textile mills and assign them equally to the intervention or the control arm by stratified randomization. Stratification will be according to the total number of employees at the mill (≤ 250/> 250) and occupational health and safety measures in place at baseline (as assessed by a walk-through survey and categorized as good or poor). Within strata, allocation to intervention or control will be through random numbers generated at Imperial College London and shared with the research team in Pakistan. Blinding of mill staff will not be possible but we will “blind” the data collection team including the technicians measuring dust levels, and the laboratory personnel involved in weighing filters for gravimetric analysis.

### Intervention

**Fig. 1:**
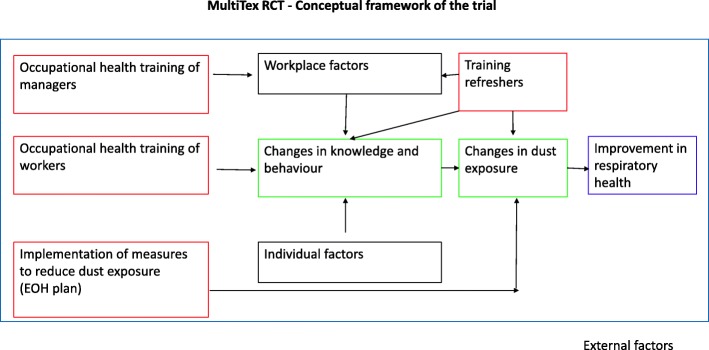
MultiTex RCT – Conceptual framework of the trial

The study’s framework (Fig. 1) describes the links between the components of the intervention. The training component is partly adapted from Robson et al.’s theory of how training can be effective in leading to improved health outcomes [20]. This considers worker and manager training as part of the wider workplace environment, where its effectiveness is modulated by other individual and organizational factors. In accordance with that framework, the intervention package will comprise four components:

1. Initial training for all workers and managers on occupational health and safety
2. Follow-up refresher sessions every 3 months
3. Formation of workplace committees (including worker representatives) to draw up, agree and promote a health and safety plan that includes wet mopping, safe disposal of cotton dust, and the use of simple face-masks, as well as further publicity about the risks from cotton dust
4. Provision of adequate supplies of face-masks to support the health and safety plan

An initial series of training sessions will be conducted in the intervention arm to explain the health hazards of cotton dust and appropriate preventive measures. These sessions will be based on ‘adult learning theory’ [18, 23], and will be delivered separately to the workers and managerial staff. To reinforce the content, regular refresher sessions will then be conducted every 3 months for the workers and every 6 months for the managers.

At each mill in the intervention arm, an Environmental and Occupational Health (EOH) plan will be developed with management and worker representatives, covering steps to be taken in implementing the intervention package. As part of the plan, wet mopping and safe disposal of cotton dust (such as keeping it in closed containers and avoiding direct contact of workers with trash) will be promoted. Guidance will be provided to ensure that any available local exhaust ventilation system is functioning satisfactorily. In addition, better personal and workplace hygiene will be promoted among the workforce. Implementation of the EOH plan will be documented as part of the trial’s fidelity assessment.

Smooth delivery of the EOH plan will be assisted by formation of a “MultiTex” Committee at each mill in the intervention arm. This Committee, which will include workers’ representatives, will be responsible for quality assurance and addressing day-to-day challenges.

In addition to training sessions, workers will be provided with simple disposable face-masks, commonly known as “surgical face-masks,” for use during working hours. MultiTex Committee members will be responsible for keeping a log of the number of face-masks distributed per shift and the number of workers who actually use them.

We do not intend to provide any intervention to the mills in the control group during the course of the main study. However on conclusion of the study, and if the trial is successful in achieving the desired outcomes, those mills will be provided with the same intervention. This will comprise all components of the intervention package except for the refresher sessions.

### Measurements and their frequencies

**Fig. 2:**
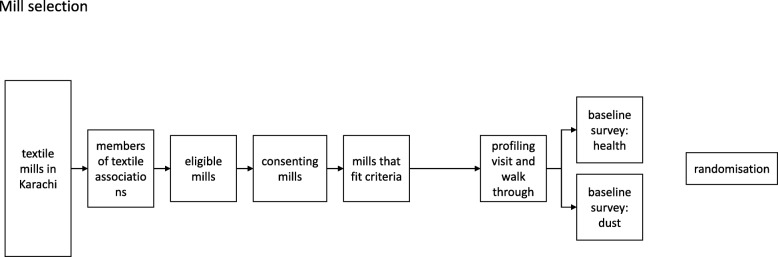
Mill selection

**Fig. 3:**
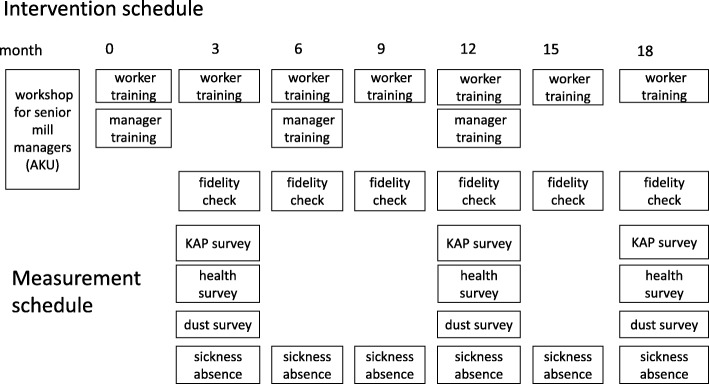
Intervention and measurement schedule; *AKU* Aga Khan University, *KAP* knowledge, attitude and practices

**Fig. 4:**
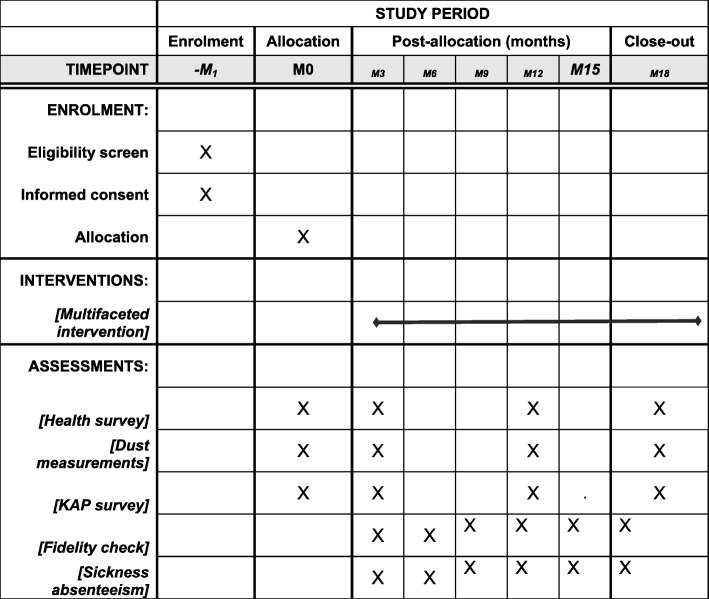
Standard Protocol Items: Recommendations for Interventional Trials (SPIRIT) Figure. Schedule of enrollment, interventions and assessments in the MultiTex RCT. *KAP* knowledge, attitude and practices

To assess the impact of the intervention, surveys will be carried out across all mills at baseline (i.e., before the intervention is implemented), and then at follow-up 3, 12 and 18 months later (Figs. 2, 3 and 4, and Additional file 1). The surveys will include detailed interviews using a questionnaire, and spirometry for each worker. In addition, personal dust exposures, measured with Institute of Occupational Medicine (IOM) samplers, will be recorded for selected sub-samples of participants.

#### Interviews

To assess respiratory symptoms we will use a translated version of the Medical Research Council (MRC), UK respiratory questionnaire [24]. Questions concerning byssinosis will be taken from the World Health Organization (WHO) respiratory questionnaire from technical report series 684 [25]. To assess knowledge, attitudes and practices (KAP) among textile workers and managers we will use a structured questionnaire which was developed by the authors as part of previous research on textile workers. The study questionnaires will be pretested and delivered by trained data collectors in the field. The total time required for the questionnaires will be approximately 20 min.

#### Spirometry and anthropometric measurements

We will use the ndd (EasyOne) spirometer to measure lung function, following established guidelines [26]. Forced expiratory volume in the first second (FEV_1_), forced vital capacity (FVC) and the FEV_1_/FVC ratio will be recorded. Pre- and post-bronchodilator spirometry will be conducted at least 1 h after the worker has started a shift (i.e., after he has been exposed to cotton dust that day). We will note the time of the day when spirometry is carried out, as well as the interval since start of the shift, and the number of consecutive days that have been worked leading up to the measurement. Every effort will be made to undertake spirometry at each follow-up on the same working day and during the same shift as at baseline. For a sub-sample of 100 randomly selected workers in the highly exposed job categories (helpers/cleaners and machine operators), pre- and post-shift spirometry will additionally be performed, to gauge cross-shift changes in lung function. We will record height in centimeters using a stadiometer, and weight in kilograms.

#### Cotton-dust exposures

We will use IOM sampling heads with Casella Apex2 pumps for personal monitoring of dust exposures, measuring the inhalable fraction of particulate matter (PM < 100 μm) over an 8-h shift. Pre- and post-sampling airflow and sampling time will be recorded, as well as temperature and humidity readings. Each individual’s average PM exposure concentration will be calculated from the difference in filter weights and sampled air volume. Individual data will then be combined to derive mean exposures for combinations of job and section at each mill. Where possible, the same workers will be monitored at follow-up as at baseline, but those who cannot be re-contacted will be replaced by others with similar job tasks. Dust sampling will be done at all the textile mills across the intervention and control arms at baseline and will be repeated at 3, 12 and 18 months after intervention. In addition to personal sampling, area sampling will be carried out in the spinning and weaving sections of each mill using a Dylos DC 1700 particle counter to measure the average concentration of dust (particle size > 0.5 or > 0.25 μm) over an 8-h shift. The particle counters will be placed at fixed sites in the centres of work stations in the spinning and weaving sections, at approximately 1.5 m height, and away from the ventilation outlets or fans. Side-by-side gravimetric and Dylos measurements will be made for a small number of sites to provide a calibration factor for the Dylos particle concentration in relation to cotton dust.

#### Qualitative assessment and economic evaluation

At the end of the study, walk-through surveys will provide direct qualitative assessment of any changes in safety provisions during the trial.

During the course of the study, we will also collect information from mills which receive the intervention on costs of its implementation and any direct or indirect savings that may have resulted. We will also document changes in staff turnover, sickness absenteeism and associated costs. Finally, if the intervention proves to be successful we will undertake a simple cost-effectiveness analysis to inform its delivery and value at a broader level.

### Study outcomes

The primary outcomes of the trial at 18 months post intervention, will be: (1) personal dust exposure levels (in mg/m^3^), and their change from baseline, in the intervention as compared with the control arm; (2) dust levels in different sections of the mill (in mg/m^3^), and their changes from baseline, in the intervention as compared with the control arm; (3) changes from baseline in the prevalence of respiratory symptoms, in the intervention as compared with the control arm (a composite variable will be used for presence of one or more respiratory symptoms including; cough, sputum, wheeze, shortness of breath and chest tightness) and (4) changes in FEV_1_ from baseline to follow-up, in the intervention as compared with the control arm.

### Process evaluation

**Fig. 5:**
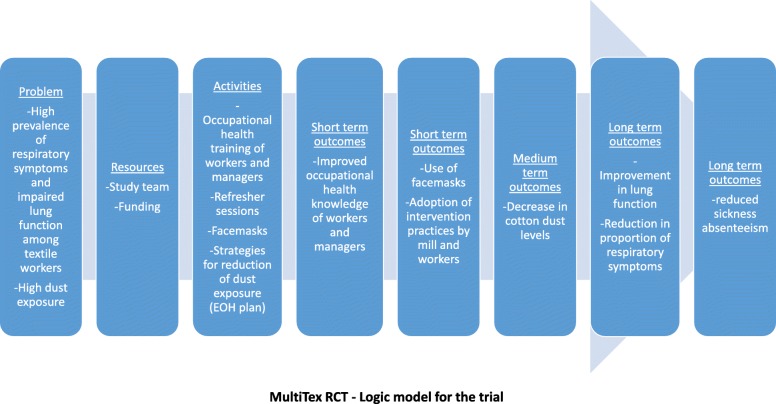
MultiTex RCT– Logic model of the trial

Process evaluation will be performed according to the logic model of the trial (Fig. 5) using the process measures below:

- For the workers’ training component: number of training sessions held; number of participants in each training session
- For improvement in KAP of workers: improvements in the proportions of workers reporting good knowledge, appropriate attitude and appropriate protective practices in the intervention compared to the control arm
- For the managers’ workshops: number and proportion of managers trained at each mill; proportion of mill managers using personal protective equipment (PPE) during visits to high-exposure areas
- Changes in KAP of managers: documented through a questionnaire and compared between the intervention and the control arm
- For administrative measures: number of meetings held by the “MultiTex” Committees; plans and policies developed at each mill as a consequence of the study; incorporation of training program in overall OHS plans of the mills
- For PPE component: number of face-masks distributed on a daily basis and proportion of workers using face-masks at random visits conducted by the MultiTex Committee members

We will use a checklist (Additional file 2) for evaluating the fidelity of our trial in line with the MRC guidelines for complex interventions, and the National Institutes of Health Behaviour Change Consortium’s (NIHBCC’s) guidelines on assessing fidelity in public health trials [27, 28]. This checklist considers aspects of fidelity in five specific domains: study design, training of providers, treatment delivery, treatment receipt, and treatment enactment. We will consider fidelity both at the mill and the worker level.

### Statistical analysis

Data will be entered through Epi Data and analyzed with STATA. Frequency counts will be derived for baseline socio-demographic variables and respiratory symptoms (categorical variables). Measures of central tendency and dispersion will be reported for continuous variables (cotton-dust levels and lung function indices). Correlations between the cotton-dust levels and lung function (FEV_1_, FVC and FEV_1_/FVC ratio) will be assessed through Pearson’s correlation coefficient.

Models based on generalized estimating equations (GEEs) will be developed to determine changes in the cotton-dust levels, frequency of respiratory symptoms, FEV_1_ (lung function indices) and respiratory sickness absenteeism. The changes in these outcome variables from baseline will be compared between the intervention and control groups. The GEE models will take into account the clustering by mills, as well as the correlation between repeated measures. Multicollinearity will be assessed between covariates and will be taken into account in the multivariable models.

We will analyze by intention to treat, and workers will be classified according to their assigned treatment group at the time of randomization. Sensitivity analyses will be performed to explore the potential impact of losses to follow-up.

### Statistical power

Based on previous occupational trials, we assumed that the intra-class correlation coefficients for the outcome measures were 0.02 [29, 30].

For cotton-dust levels: assuming the level of significance at 5%, power 80% and a 15% or 0.65 mg/m^3^ difference in mean PM levels in the intervention arm compared to the control arm (expected mean dust level in control arm: 0.6 with a standard deviation of 2.5); the sample size required without clustering is 233/arm. The variance inflation factor is:

$$ \left[1+\left(50-1\right)\ast 0.02\right)\Big]=1.98, $$

so the sample size is inflated to 461, which rounds up to 10 groups of 50 in each arm – which increases to 10 groups of 61 in each arm after inflating for losses (* 1.05 * 1.05 * 1.1 = * 1.212).

For respiratory symptoms: the trial is powered to detect a difference of 10% in the proportion of workers reporting respiratory symptoms between the intervention and control arms. Assuming a level of significance at 5%, power 80%, the proportion of workers having respiratory symptoms at the baseline as 40%; without clustering we would require 354/arm. With clustering, the new sample size is 701/arm which – in groups of 50 – means 14 groups of 50 in each arm of the study. Inflating these numbers for losses gives 14 groups of 61.

For lung function: assuming level of significance at 5%, power 80%, 5% or a 150-ml difference in mean FEV_1_ (standard deviation 31) in the intervention arm compared to the control arm (expected mean FEV_1_ in control arm: 3279 ml, standard deviation 690); the requirement without clustering would be 333 per arm, which inflates to 660 with clustering, which again leads to 14 groups of 50 in each arm and this again inflates to 14 groups of 61 after inflating for losses.

Based on the above calculation, we intend to recruit 854 workers from 14 clusters in each arm, a total of 1708 participants in 28 clusters.

### Ethical considerations

The study has received full ethical approval from the Ethics Review Committee at Aga Khan University, Pakistan (2019-0962-3710) and the Research Ethics Committee at Imperial College London, UK (19IC4968), as well as the Research Ethics Committee of the National Bioethics Committee in Pakistan (4-87/NBC-402/19/483). Written informed consent will be obtained from each participant, and they will be free to leave the study whenever they desire. The workers’ workshops will be brief and conducted in small groups so that mill productivity is not affected. A small financial compensation will be provided to each participant at each time of data collection. Workers will be provided with light refreshments after spirometry and during workshops.

All the participants will be informed verbally about their spirometry results and guided accordingly. If a study participant wishes to receive the spirometry report this will be provided. Workers who are found to have significant, previously unrecognized lung disease will be counseled, and if they wish, they will be referred to a suitable government hospital for further evaluation and management. A letter of referral will be provided.

All data will be kept confidential and the questionnaire reports and spirometry results of individual workers will not be shared with their employers. However, employers will be provided with anonymized summary results for their mills at the end of the study to help them plan appropriate control and preventive measures.

The study should help the textile workers to improve their knowledge about the health hazards of cotton-dust exposure and guide researchers further towards developing strategies for the implementation of protective measures. In addition, the textile workers will be tested for their respiratory function through spirometry, which will be free of cost. Those in the intervention arm will also be provided with free disposable face-masks on a daily basis during the study period.

Incentives for the workplaces will include free monitoring of dust levels, employee health assessments and training. Such measures will benefit their audit and compliance processes, and in this way they may be able to get better business by fulfilling the requirements of major textile buyers globally [31]. After the conclusion of this study, by agreement before the trial, expert advice on OHS improvements will also be provided to the mills in the control arm.

### Dissemination

In Pakistan, dissemination seminars will be organized to share findings with various stakeholders including representatives from textile mill associations, concerned non-governmental organizations (NGOs), national and provincial Environmental Protection Agencies (EPAs), Labor Department, Ministry of Textile and Ministry of Health. Textile mill and business associations are key stakeholders for this project. They will help in getting access to the industry and will also facilitate the implementation of policy recommendations that emerge from the research.

## Discussion

This trial will test whether a simple, low-cost intervention package can reduce dust levels in textile mills, and improve the respiratory health of textile workers in developing countries such as Pakistan. The randomized controlled design of the project should provide robust scientific evidence regarding the effectiveness of such an intervention and, if it is found to be successful, will have significant implications for similar settings in Pakistan and elsewhere.

We chose a cluster randomized design since the intervention will have some impact at the individual level, but more so at the cluster level, through changes in the management and the overall organizational plans and practices. Because much of the effect may be at the level of the plant, it would not be possible to randomize individuals.

We will measure the inhalable fraction (≤ 100 μm) of dust at the textile mills in accordance with the Health and Safety Executive (HSE), UK guidelines [32]. The smaller dust particles (≤ 2.5 μm; respirable fraction) are known to be more hazardous compared to the larger ones; however, previous studies demonstrated the feasibility of using the inhalable fraction for assessing cotton-dust exposure among textile workers [33]. Moreover, measurement of the inhalable fraction will allow comparison with cotton-dust standards. Due to logistic and financial constraints, measurement of bacterial endotoxin levels will not be possible in this trial, but we believe that cotton-dust measurements will provide a reasonable approximation of the endotoxin levels and related health effects in Pakistani textile mills [33, 34].

Recruitment of the required number of mills and workers will be challenging. The study team is already in contact with stakeholders in the textile industry in Pakistan and is disseminating wider publicity of the study in Karachi and networking with relevant business associations for better access to textile mills. In order to access textile workers for data collection and training, incentives will be provided for workers, including free spirometry and a small financial compensation for their time.

Several limitations may need to be considered when interpreting the findings.“Contamination” of the control sites might occur if contact with participants or mill representatives from the intervention arm led to the adoption of some of the intervention by mills or workers in the control arm. However, any such contamination should be limited since there are limited opportunities for textile workers in Pakistan to unionize or socialize as a group. Moreover, we will ask the mill management to keep their participation status confidential from other mills until the study has been completed.

Even in the absence of contamination, however, study participants and mill representatives in the control arm of the study may alter their behaviour or introduce OHS initiatives, simply because they know that they are being observed (a Hawthorne Effect). This would tend to dilute the estimated effectiveness of the intervention. We will try to reduce any such bias by providing minimal information on the intervention to the control group. In addition, any changes over the course of the study in the OHS provisions at control mills will be documented, and taken into account in interpretation.

Loss to follow-up may be another problem. Labor turnover is generally high in Pakistani textile mills and workers may also change sections within the same mill. If the worker has switched job to another section within the same mill we will try to approach him for follow-up but if he has moved to another mill, we may not be able to follow him. Where a worker is not able to continue working due to sickness, we will note the information. Loss to follow-up may also occur due to death or outmigration (to another city), although this is less likely. Such losses are inevitable but have been taken into account in setting the sample size.

Despite these challenges, we believe that the study had the potential to generate useful information that may lead to cost-effective policies for the prevention of work-related respiratory disease among textile workers in developing countries.

### Trial status

Recruiting

## Supplementary information

**Additional file 1.** Standard Protocol Items: Recommendations for Interventional Trials (SPIRIT) 2013 Checklist: recommended items to address in a clinical trial protocol and related documents.

**Additional file 2.** Intervention fidelity checklist – MultiTex RCT.

## Data Availability

Not applicable

## Abbreviations

EOH: Environmental and Occupational Health
EPA: Environmental Protection Agency
FEV_1_: Forced expiratory volume in first second
FVC: Forced vital capacity
GDP: Gross Domestic Product
GEE: Generalized estimating equations
HSE: Health and Safety Executive, UK
IOM: Institute of Occupational Medicine
KAP: Knowledge, attitude and practices
MRC: Medical Research Council, UK
NIHBCC: National Institutes of Health Behaviour Change Consortium
OHS: Occupational health and safety
PM: Particulate matter
PPE: Personal protective equipment
WEL: Workplace exposure limits
